# Coexistence of cerebral hypometabolism and neuroinflammation in the thalamo-limbic-brainstem region in young women with functional somatic syndrome

**DOI:** 10.1186/s13550-020-00617-1

**Published:** 2020-03-20

**Authors:** Takashi Matsudaira, Tatsuhiro Terada, Tomokazu Obi, Masamichi Yokokura, Yukitoshi Takahashi, Yasuomi Ouchi

**Affiliations:** 1 Department of Biofunctional Imaging, Preeminent Medical Photonics Education & Research Center, Hamamatsu University School of Medicine, 1-20-1 Handayama, Higashi-ku, Hamamatsu, 431-3192 Japan; 2 Department of Neurology, Shizuoka Institute of Epilepsy and Neurological Disorders, NHO, National Epilepsy Center, Shizuoka, Japan; 3 Department of Psychiatry, Hamamatsu University School of Medicine, Hamamatsu, Japan; 4 Department of Pediatrics, Shizuoka Institute of Epilepsy and Neurological Disorders, NHO, National Epilepsy Center, Shizuoka, Japan

**Keywords:** [^11^C]DPA713, [^18^F]FDG, Functional somatic syndrome, Glucose metabolism, Neuroinflammation, Positron emission tomography

## Abstract

**Background:**

Functional somatic syndrome (FSS) is a disorder characterized by clusters of medically unexplained symptoms. Some women suffer from persistent FSS after human papillomavirus (HPV) vaccination. However, a causal relationship has not been established, and the pathophysiology of FSS remains elusive. Here, we aimed to identify the brain regions showing altered cerebral metabolism and neuroinflammation in patients with FSS and to correlate the measures of positron emission tomography (PET) with clinical data. Twelve women diagnosed with FSS following HPV vaccination (FSS group) underwent both [^18^F]FDG-PET to measure glucose metabolism and [^11^C]DPA713-PET to measure neuroinflammation. [^18^F]FDG standardized uptake value ratio (SUVR) and [^11^C]DPA713 binding potential (BP_ND_) values were compared voxel-wise between the FSS and control groups (*n* = 12 for [^18^F]FDG, *n* = 16 for [^11^C]DPA713). A region-of-interest (ROI)-based analysis was performed to correlate PET parameters with clinical scores. Statistical significance was set at *p* < 0.05 corrected for multiple comparisons.

**Results:**

Statistical parametric mapping revealed a concomitant significant decrease of [^18^F]FDG SUVR and increase of [^11^C]DPA713 BP_ND_ in the regions covering the thalamus, mesial temporal area, and brainstem in the FSS group. Correlation analysis revealed that intelligence and memory scores were significantly positively correlated with [^18^F]FDG SUVR and negatively so with [^11^C]DPA713 BP_ND_ in these regions. A direct comparison between [^18^F]FDG SUVR and [^11^C]DPA713 BP_ND_ revealed a significant positive correlation in the right hippocampus and amygdala.

**Conclusions:**

Cerebral hypometabolism with neuroinflammation occurring in the thalamo-limbic-brainstem region may reflect the pathophysiology of FSS.

## Background

Functional somatic syndrome (FSS) is a disorder characterized by clusters of medically unexplained symptoms even after extensive examinations [1]. Most well-known FSS conditions are fibromyalgia, chronic fatigue syndrome (CFS), and irritable bowel syndrome, which often exhibit several common symptoms and share similar treatments [1, 2]. Recent studies from Japan and Denmark have reported that some women exhibit various long-lasting physical, psychological, cognitive, and behavioral symptoms following human papillomavirus (HPV) vaccination (a prophylactic measure against future development of cervical cancer) [3, 4]. In Japan, the post-vaccination symptoms have finally regarded as various somatic symptoms without the causal relationship with HPV vaccination [5]; however, the pathophysiology of FSS itself remains elusive.

FSS symptoms, such as widespread pain, psychiatric symptoms, and cognitive dysfunction, suggest a disturbance of the somatosensory system based on the thalamus, a relay region for almost all sensory information, and the limbic system, a part of the brain involved in memory, emotion, and the default-mode network [6]. Thus, we hypothesized that the etiology of FSS may be associated with the disruption of the brain milieu in these regions.

Positron emission tomography (PET) with [^18^F]FDG has been extensively used to detect cerebral glucose metabolic alterations, reflecting cerebral function (neuronal and synaptic activity) and/or cellular abnormalities [7]. Recent studies indicated that a disturbance of the brain milieu accompanies neuroinflammation [8, 9], which can be visualized by PET with radiotracers for the 18 kDa translocator protein (TSPO) developed in glial cells such as microglia and astrocytes [8, 10–12]. Among radiotracers for TSPO, [^11^C]DPA713 is considered a second-generation ligand for the TSPO. Several studies on FSS implicated changes at the immunomolecular level, suggesting the involvement of neuroinflammation in the pathophysiology of FSS [13–15]. While previous PET studies revealed regional cerebral abnormalities in fibromyalgia and CFS [16–18], there are no pathophysiological studies on FSS regarding symptom-related brain regions with simultaneous cerebral glucose metabolism and neuroinflammation.

Therefore, we aimed to identify the brain regions showing altered cerebral metabolism and neuroinflammation in patients with FSS and to correlate the results of PET with clinical data to better understand the pathophysiology of FSS.

## Material and methods

### Participants

From February 2014 to February 2017, women who complained of symptoms of unknown cause after HPV vaccination were admitted to the Shizuoka Institute of Epilepsy and Neurological Disorders in Japan. The diagnosis of FSS was based on the various persistent somatic symptoms after HPV vaccination, which remained medically unexplained even after extensive medical assessment or overlapped with CFS and fibromyalgia [1]. The exclusion criteria were as follows: the presence of pathological findings on magnetic resonance imaging (MRI) unrelated to symptoms, a history of neurological and psychiatric disorders prior to FSS onset, and organic diagnosis during symptom development. After applying the exclusion criteria, 12 female patients with FSS were included in this study (FSS group; mean age ± standard deviation [SD] 19.3 ± 1.5 years). The FSS group underwent both [^18^F]FDG-PET to measure glucose metabolism and [^11^C]DPA-713 PET to measure neuroinflammation. A total of 28 healthy women were selected as controls; they had no neurological problems, history of serious illness, or psychiatric disorders. Of the 28 controls, 12 underwent [^18^F]FDG-PET (FDG control group; mean age 32.8 ± 9.5 years); the other 16 were age-matched and underwent [^11^C]DPA713-PET (DPA control group; mean age 20.6 ± 1.6 years).

The clinical scores for the FSS group were assessed using the following questionnaires: numerical rating scale for pain, Chalder fatigue scale, Stanford sleepiness scale, Wechsler Adult Intelligence Scale-Third Edition (WAIS-III), and Wechsler Memory Scale-Revised (WMS-R). The numerical rating scale for pain ranges from 0 (no pain) to 10 (worst pain) [19]. The Chalder fatigue scale consists of 11 questions, and the total score ranges from 0 to 33 [20], with higher scores indicating a greater degree of fatigue. The Stanford sleepiness scale evaluates sleepiness ranging from 1 (wide awake) to 7 (no longer fighting sleep) [21]. The WAIS-III and WMS-R evaluate cognitive ability, with mean IQ and memory scale defined as 100 and standard deviation as 15.

This study was reviewed and approved by the Ethics Committee of Hamamatsu PET Medical Photonics Center and the Shizuoka Institute of Epilepsy and Neurological Disorders. We obtained written informed consent from all patients or their guardians prior to PET scan.

### MRI scanning

Prior to PET scanning, brain MRI (1.5-T GE, Signa Excite HDx, GE Medical Systems, Milwaukee, WI) was performed with three-dimensional mode sampling to determine the areas for setting region of interest (ROI), using the following acquisition parameters: repetition time = 25.0, echo time = minimum, flip angle 30°, slice thickness 1.5 mm, matrices 256 × 128, and field of view = 24.0. With reference to the measures of tilt angle and spatial coordinates obtained to determine the anterior commissure–posterior commissure (AC–PC) line on each participant’s sagittal MRI, a PET gantry was set as parallel to the AC–PC line by tilting and moving the gantry for each study. The MRI measurements and mobile PET gantry allow us to reconstruct PET images parallel to the AC–PC line without reslicing.

### PET data acquisition

All participants underwent a series of PET measurements after brain MRI scanning. We used a high-resolution brain PET scanner (SHR12000; Hamamatsu Photonics K.K., Hamamatsu, Japan). The gantry was set parallel to the AC–PC line determined by brain MRI. A thermoplastic face mask was used to fix the head to the same place during each PET scan. First, a 10-min transmission scan with a ^68^Ge/^68^Ga source was performed for attenuation correction. Then, a PET measurement with [^11^C]DPA713 was performed to investigate neuroinflammation on both the FSS and DPA control groups. In this measurement, serial emission scans were obtained for 90 min after a slow bolus venous injection (taking 1 min) of a 5 MBq/kg dose of [11C]DPA713. Lastly, a static 15-min PET scan was done 45 min after injection of a 100-MBq dose of [^18^F]FDG. The interval of these scans was 2.5 h after the cessation of the [^11^C]DPA713 measurement. We recruited age-matched control female participants in the DPA control group for comparison because age is a key factor for neuroinflammation in the brain [22]. No arterial sampling was performed along with each PET measurement.

### Image data processing

To measure glucose metabolism, a semi-quantitative ratio index of [^18^F]FDG was calculated as standardized uptake value (SUV) ratio, where the SUV represents tracer activity per injected dose normalized to body weight. The SUV of each region was divided by the SUV of the whole brain as the global mean in each participant, and expressed as the SUV ratio (SUVR) image.

The non-displaceable binding potential (BP_ND_) of [^11^C]DPA-713 was estimated with the simplified reference tissue model using the PMOD 3.4 software (PMOD Technologies Ltd., Zurich, Switzerland) based on supervised cluster analysis, as described previously [22]. In short, we first generated a normalized input curve by averaging the time activity curves (TAC) from areal ROIs placed over the bilateral frontal, temporal, parietal, and occipital cortices; thalamus; putamen; caudate; cerebellar hemisphere; and brain stem. Then, this normalized mean TAC adjusted to the TAC with lowest PET counts from the cerebral cortical regions in each participant was used as the TAC for the reference input curve of the patients and controls. The lowest TAC for each participant was determined using clustered analysis, in which small circular ROIs of 2 mm in diameter were placed fully within the ROIs already determined in the cerebral cortical and subcortical regions on MRI, and the TACs with the lowest peak from each TAC of all of the small ROIs was obtained. The extracerebral structures were then masked by demarcating the cerebral regions on the brain MRI. Finally, the [^11^C]DPA713 BP_ND_ was obtained for each region and expressed as the BP_ND_ image.

### Statistical parametric mapping analysis

To identify the regions of significantly decreased glucose metabolism and elevated neuroinflammation in the FSS group, the voxel-based analyses of the [^18^F]FDG SUVR and [^11^C]DPA713 BP_ND_ image maps were analyzed using the Statistical Parametric Mapping 8 software (SPM8; Wellcome Department of Imaging Neurosciences, London, UK). All [^18^F]FDG SUVR and [^11^C]DPA713 BP_ND_ images were first normalized to the Montreal Neurological Institute (MNI) 152 standard space provided by SPM8 used as a default template. The between-group comparisons (FSS group vs. FDG control group and FSS group vs. DPA control group) for each parameter were performed using family-wise error (FWE) multiple-comparisons correction, with the statistical threshold set at *p* < 0.05.

To investigate the change in regional brain volume between FSS and control (age-matched DPA control) groups, voxel-based morphometry (VBM) was performed using SPM8 with Diffeomorphic Anatomical Registration Through Exponentiated Lie Algebra (DARTEL) toolbox. T1-weighted images were segmented into gray matter, white matter, cerebrospinal fluid, skull, soft tissue, and air/background using the “New Segment” algorithm in SPM8. A customized template was created from the images of FSS and control groups. Modulated gray and white matter segments were normalized to Montreal Neurological Institute (MNI) 152 standard space default template provided by SPM8. Images were smoothed with 8-mm FWHM Gaussian kernel and two-sample *t* tests throughout the whole brain were performed in SPM8. A voxel-level height threshold was set at *p* < 0.05 (FWE corrected).

### ROI analysis

Regional [^18^F]FDG SUVR and [^11^C]DPA713 BP_ND_ values were determined from ROIs located in 10 brain regions: the pons, midbrain, bilateral hippocampus, amygdala, thalamus, and anterior cingulate cortices on the individual MRI because a previous study had demonstrated the presence of neuroinflammation in these areas in patients with CFS, a type of FSS [18]. These ROIs were then automatically transferred onto corresponding [^18^F]FDG SUVR and [^11^C]DPA713 BP_ND_ parametric images. Regional [^18^F]FDG SUVR and [^11^C]DPA713 BP_ND_ values derived from these ROIs were determined using the PMOD software. We investigated the relationship between the clinical scores and each of the above-mentioned regional [^18^F]FDG SUVR and [^11^C]DPA-713 BP_ND_ values using Pearson’s correlation analysis and linear regression analysis because the normality was found for these clinical data. Statistical significance was set to *p* < 0.005 (*p* < 0.05 with Bonferroni’s correction). Furthermore, we focused on these regions to examine whether and where neuroinflammation was directly associated with glucose hypometabolism through correlations between [^18^F]FDG SUVR and [^11^C]DPA-713 BP_ND_ values from each ROI in the FSS group using Pearson’s correlation analysis and linear regression analysis in the GraphPad Prism 5.0 package (GraphPad, San Diego, CA, USA). Statistical significance was set to *p* < 0.005 (*p* < 0.05 with Bonferroni’s correction).

## Results

### Demographic and clinical characteristics of the study participants

The demographic and clinical characteristics, including the numerical rating scale for pain, the Chalder fatigue scale, Stanford sleepiness scale, WAIS-III, and WMS-R scores, are presented in Table 1. All groups were matched for sex. The mean ages of the DPA control group (mean age ± SD 20.6 ± 1.6) and the FSS group (19.3 ± 1.5 years) were not statistically different, but the FDG control group (32.8 ± 9.5 years) was significantly older than the FSS group. In the WMS-R test, there were some missing scores due to lack of persistence in some patients.

**Table 1:** Demographic and clinical characteristics of the study participants

Characteristics	FSS group (*n* = 12)	Control group	
		[^18^F]FDG PET (*n* = 12)	[^11^C]DPA713 PET (*n* = 16)
Age (years)	19.3 ± 1.5	32.8 ± 9.5*	20.6 ± 1.6
Women/men	12/0	12/0	16/0
Disease duration (years)	4.3 ± 0.7	n.a.	n.a.
Clinical scores (score range)		n.a.	n.a.
Numerical rating scale (0 - 10)^a^	4.0 ± 1.8	n.a.	n.a.
Chalder fatigue scale (1 - 33)^b^	21.4 ± 7.5	n.a.	n.a.
Stanford sleepiness scale (1 - 7)^c^	3.4 ± 1.4	n.a.	n.a.
WAIS-III^d^			
Full-scale IQ	80.6 ± 11.6	n.a.	n.a.
Verbal IQ	81.8 ± 8.6	n.a.	n.a.
Performance IQ	83.7 ± 16.8	n.a.	n.a.
Verbal comprehension	86.7 ± 9.3	n.a.	n.a.
Perceptual organization	83.6 ± 16.4	n.a.	n.a.
Working memory	79.6 ± 14.2	n.a.	n.a.
Processing speed	80.4 ± 18.4	n.a.	n.a.
WMS-R^d^			
Full memory	86.1 ± 16.6	n.a.	n.a.
Verbal memory	85.7 ± 19.2	n.a.	n.a.
Visual memory	87.9 ± 16.2	n.a.	n.a.
Attention memory	77.3 ± 15.2	n.a.	n.a.
Delayed memory	74.7 ± 20.6	n.a.	n.a.

Data are the mean ± standard deviation (SD) or the number

*IQ* intelligence quotient, *n.a*. not available, *WAIS-III* Wechsler Adult Intelligence Scale-Third Edition, *WMS-R* Wechsler Memory Scale-Revised

*p < 0.05

^a^Pain scale ranges from 0 (no pain) to 10 (worst pain)

^b^Fatigue scale with higher scores indicating a greater degree of fatigue

^c^Sleep scale ranges from 1 (wide awake) to 7 (no longer fighting sleep)

^d^The average IQ and memory score is defined as 100 and the SD as 15 (i.e., approximately two-thirds of all scores between 85 and 115)

### SPM analysis: comparison of [^18^F]FDG SUVR and [^11^C]DPA713 BP_ND_ between the FSS and control groups

**Fig. 1:**
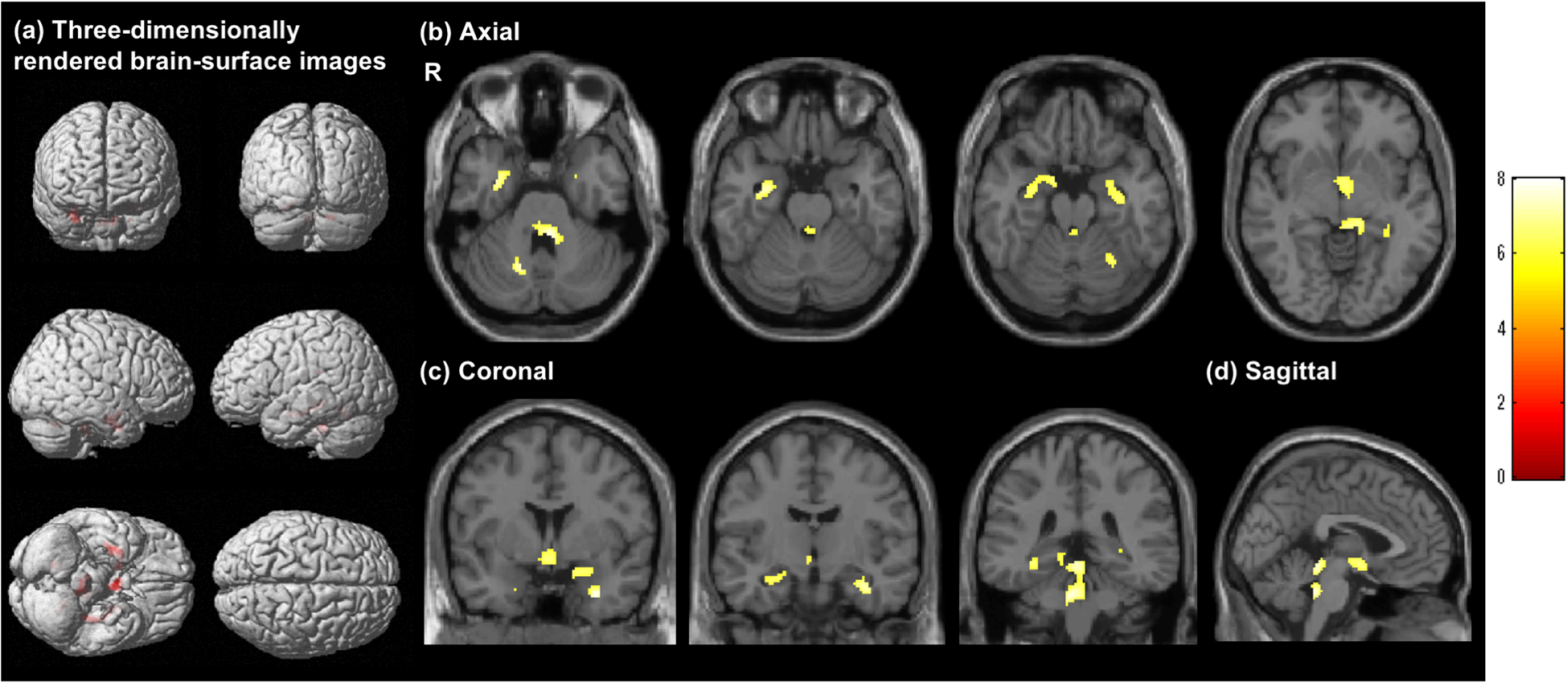
Voxel-wise analysis of [^18^F]FDG standardized uptake value ratio images (*p* < 0.05, family-wise error corrected). Regions with a significant reduction of [^18^F]FDG standardized uptake value ratio (SUVR) are highlighted on three-dimensionally rendered brain-surface images (**a**). Axial (**b**), coronal (**c**), and sagittal (**d**) magnetic resonance images show the regions with a significant reduction of [^18^F]FDG SUVR (*p* < 0.05, family-wise error corrected). The color bar denotes the *T* value (0–8). R, right

The results of SPM for [^18^F]FDG SUVR in the FSS group compared with FDG control group are shown in Fig. 1. On three-dimensionally rendered images, statistically significant differences of [^18^F]FDG SUVR were observed in the mesial temporal region, thalamus, circumventricular region, and tegmentum of the brainstem, bilaterally (Fig. 1a, Table 2a). Although there was an age difference between the FSS and FDG control groups, this finding indicated that the brain regions with reduced cerebral glucose metabolism were mostly confined to the thalamo-limbic-tegmental brainstem system in FSS.

**Table 2:** Statistical parametric mapping results for [^18^F]FDG SUVR and [^11^C]DPA-713 BP_ND_

Anatomical region	BA	Talairach coordinate			Cluster size	Voxel *T* size	Z score	Cluster *p* value		
		x	y	z				FWE	FDR	Uncorrected
a: [^18^F]FDG SUVR (FSS group vs. [^18^F]FDG PET control group)										
R uncus	36	31.7	− 1.4	− 28.5	257	8.01	5.43	< 0.001	< 0.001	< 0.001
R hippocampus		31.7	− 8.7	− 19.7		7.28	5.14			
R parahippocampus	34	17.8	− 0.8	− 15.1		6.76	4.92			
L thalamus		− 9.9	− 29.3	− 3.6	356	6.70	4.90	< 0.001	< 0.001	< 0.001
L hippocampus		− 33.7	− 31.4	− 6.8	197	6.93	5.0	< 0.001	< 0.001	< 0.001
L insula	13	− 41.6	− 31.9	21.9	7	6.80	4.94	0.019	0.473	0.368
L anterior cingulate	25	0	− 0.34	− 6.7	141	6.75	4.92	< 0.001	0.001	0.001
L uncus	28	− 21.8	2.5	− 27.0	3	6.16	4.65	0.029	0.566	0.566
b: [^11^C]DPA-713 BP_ND_ (FSS group vs. [^11^C]DPA713 PET control group)										
L thalamus		− 4.0	− 27.2	− 2.0	4560	10.18	6.41	< 0.001	< 0.001	< 0.001
L amygdala		− 25.7	− 1.2	− 23.5	208	7.78	5.54	< 0.001	< 0.001	< 0.001
L hippocampus		− 25.7	− 14.2	− 11.1		7.30	5.34			
L parahippocampus	34	− 17.8	− 6.7	− 18.2		5.72	5.07			
R inferior temporal gyrus	20	55.4	− 13.1	− 29.6	58	7.35	5.36	< 0.001	0.034	0.009
R middle temporal gyrus	20	55.4	− 39.6	− 29.6	61	7.22	5.30	< 0.001	0.034	0.008
R fusiform gyrus	20	55.4	− 34.0	− 20.2		6.90	5.15			
R middle temporal gyrus	21	49.5	4.55	− 25.5	144	7.11	5.25	< 0.001	0.001	< 0.001
L superior temporal gyrus	38	− 37.6	12.2	− 27.5	31	6.79	5.10	0.002	0.12	0.047
L fusiform gyrus	20	− 55.4	− 16.6	− 21.0	22	6.71	5.07	0.004	0.182	0.087
L superior frontal gyrus	6	− 5.9	8.9	62.2	20	6.49	4.96	0.005	0.182	0.101
L middle frontal gyrus	11	− 27.7	34.4	− 11.8	21	6.46	4.95	0.005	0.182	0.094
R insula	30	41.6	− 26.2	19.7	12	6.16	4.80	0.010	0.321	0.196

*SUVR* standardized uptake value ratio, *BP*_*ND*_ binding potential, *BA* Brodmann’s area, *L* left, *R* right, *FWE* family-wise error, *FDR* false discovery rate

**Fig. 2:**
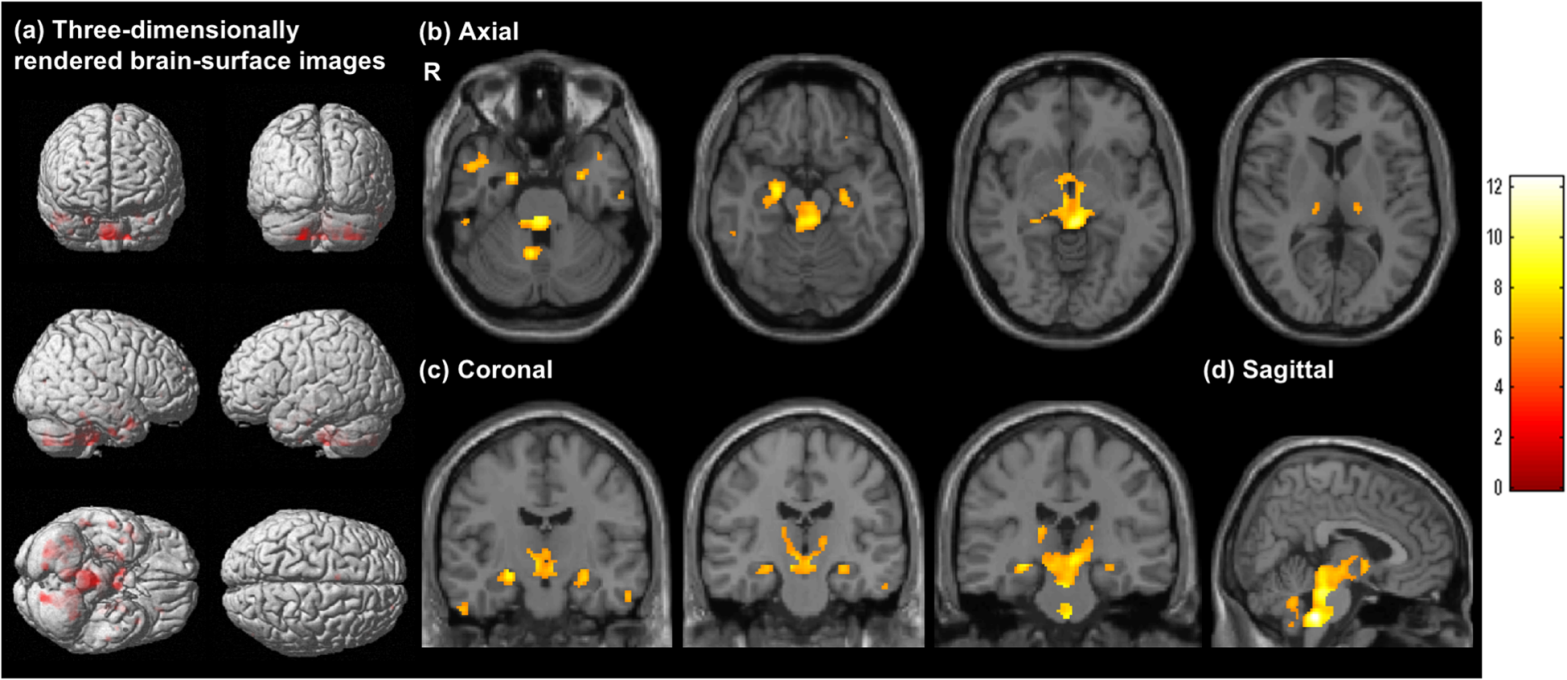
Voxel-wise analysis of [^11^C]DPA713 binding potential images (*p* < 0.05, family-wise error corrected). Regions with a significant increase of [^11^C]DPA713 binding potential (BP_ND_) are highlighted on three-dimensionally rendered brain-surface images (**a**). Axial (**b**), coronal (**c**), and sagittal (**d**) magnetic resonance images show the regions with a significant increase of [^11^C]DPA713 BP_ND_ (*p* < 0.05, family-wise error corrected). The color bar denotes the *T* value (0–12). R, right

The results of SPM for [^11^C]DPA713 BP_ND_ in the FSS group compared with DPA control group are shown in Fig. 2. On three-dimensionally rendered images, a significant increase of [^11^C]DPA713 BP_ND_ levels was observed in the mesial temporal and inferior frontal cortices, thalamus, and tegmentum of the brainstem (Fig. 2a, Table 2b). The results of SPM on reduced [^18^F]FDG SUVR and increased [^11^C]DPA713 BP_ND_ were similar in various brain regions in the FSS group, suggesting that glucose hypometabolism occurred concomitantly with neuroinflammation in key brain regions, i.e., the thalamo-limbic-brainstem region.

The VBM analysis using SPM showed no significant difference in regional brain volume between the FSS and control groups (data not shown).

### ROI analyses

#### (i) Direct relationship between [^18^F]FDG SUVR and [^11^C]DPA713 BP_ND_

**Fig. 3:**
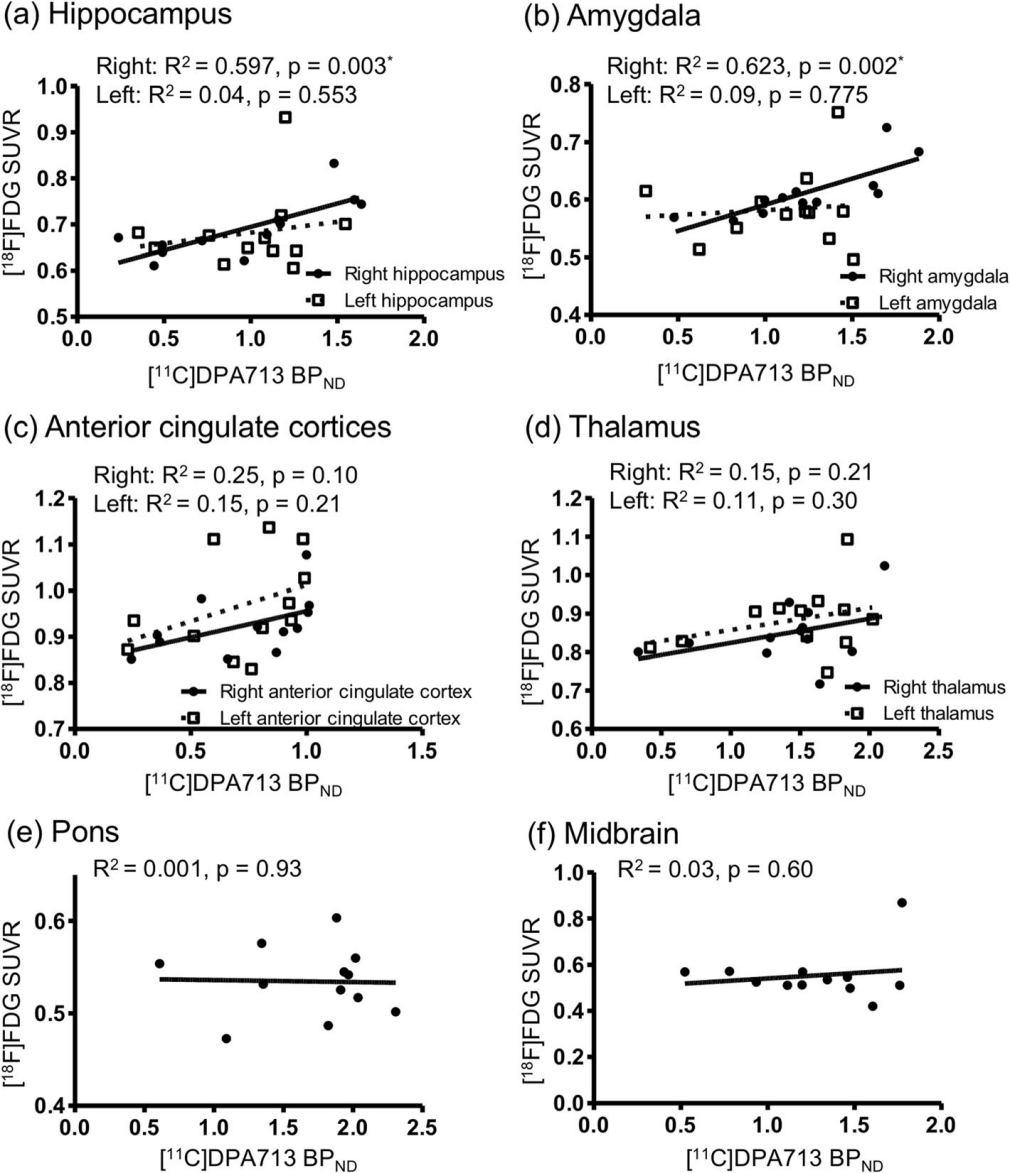
Correlations between [^11^C]DPA713 binding potential and [^18^F]FDG standardized uptake value ratio. In the right hippocampus (**a**) and the right amygdala (**b**), [^11^C]DPA713 binding potential (BP_ND_) and [^18^F]FDG standardized uptake value ratio (SUVR) were significantly positively correlated (*p* < 0.05, corrected for multiple comparisons). The anterior cingulate cortex (**c**) and thalamic regions (**d**) tended to show positive correlations between [^11^C]DPA713 BP_ND_ and [^18^F]FDG SUVR. The pons (**e**) and midbrain (**f**) shows no clear correlations between [^11^C]DPA713 BP_ND_ and [^18^F]FDG SUVR

Direct comparisons between [^18^F]FDG SUVR and [^11^C]DPA713 BP_ND_ in the same ROIs showed significantly positive correlations in the right hippocampus (*R*^2^ = 0.597, *p* = 0.003) and right amygdala (*R*^2^ = 0.556, *p* = 0.002) (Fig. 3a, b). There was no significant correlation in the anterior cingulate cortices and thalamus (Fig. 3c, d).

#### (ii) Correlations of clinical scores with [^18^F]FDG SUVR or [^11^C]DPA713 BP_ND_

Pearson’s correlation analysis revealed significantly positive correlations between the levels of [^18^F]FDG SUVR in the left thalamus (*r* = 0.81, *p* < 0.05 corrected) and working memory in WAIS-III, and between the levels of [^18^F]FDG SUVR in the left hippocampus (*r* = 0.80, *p* < 0.05 corrected) and right thalamus (*r* = 0.84, *p* < 0.05 corrected) and processing speed performance in WAIS-III in the FSS group (Table 3a). Regarding the clinical relevance on [^11^C]DPA713 BP_ND_, Pearson’s correlation analysis revealed significantly negative correlations between [^11^C]DPA713 BP_ND_ levels in the left hippocampus (*r* = − 0.77, *p* < 0.05 corrected), right thalamus (*r* = − 0.79, *p* < 0.05 corrected), and pons (*r* = − 0.81, *p* < 0.05 corrected) and verbal comprehension in WAIS-III, and between [^11^C]DPA713 BP_ND_ levels in the right thalamus and verbal memory in WMS-R (*r* = − 0.85, *p* < 0.05, corrected) (Table 3b).

**Table 3:** Correlation between [^18^F]FDG SUVR or [^11^C]DPA713 BP_ND_ values and clinical scores

Clinical score	Anatomical region	Side	Correlation coefficient (*r*)	*p* value
a: [^18^F]FDG SUVR				
WAIS-III				
Working memory	Hippocampus	R	0.65	0.022
	Thalamus	R	0.64	0.025
	Thalamus	L	0.81	0.002*
Processing speed	Hippocampus	R	0.64	0.026
	Hippocampus	L	0.80	0.002*
	Amygdala	R	0.61	0.034
	Thalamus	R	0.84	< 0.001*
	Thalamus	L	0.67	0.017
WMS-R				
Attention memory	Hippocampus	R	0.70	0.024
b: [^11^C]DPA713 BP_ND_				
WAIS-III				
Verbal comprehension	Hippocampus	L	− 0.77	0.003*
	Amygdala	L	− 0.62	0.031
	Anterior cingulate	R	− 0.71	0.009
	Anterior cingulate	L	− 0.74	0.006
	Thalamus	R	− 0.79	0.002*
	Thalamus	L	− 0.75	0.005
	Pons		− 0.81	0.001*
	Midbrain		− 0.58	0.049
WMS-R				
Verbal memory	Hippocampus	L	− 0.67	0.024
	Amygdala	L	− 0.70	0.017
	Anterior cingulate	L	− 0.66	0.027
	Thalamus	R	− 0.85	< 0.001*
	Thalamus	L	− 0.69	0.018
	Pons		− 0.73	0.010

*SUVR* standardized uptake value ratio, *BP*_*ND*_ binding potential, *L* left, *R* right, *WAIS-III* Wechsler Adult Intelligence Scale-Third Edition, *WMS-R* Wechsler Memory Scale-Revised

*p < 0.05 with Bonferroni’s correction

## Discussion

To the best of our knowledge, this is the first study to clarify the concurrent changes in cerebral glucose hypometabolism and neuroinflammation in patients with FSS. In this study, we showed that young women with FSS had significantly decreased [^18^F]FDG SUVR and increased [^11^C]DPA-713 BP_ND_ in unique brain regions covering the thalamus, mesial temporal region, and tegmentum of the brainstem. We also showed that [^18^F]FDG SUVR and [^11^C]DPA-713 BP_ND_ values in these regions were associated with clinical scores in WAIS-III and WMS-R tests. Thus, cerebral glucose hypometabolism and elevated neuroinflammatory responses in the confined brain regions together with their clinical relevance reflect the pathophysiology of FSS.

Cerebral glucose metabolism is related to the cerebral blood flow (CBF) [23], and CBF measurement by single-photon emission computed tomography (SPECT) might suggest the pathological mechanism of FSS. Indeed, our previous study using ^123^IMP-SPECT in patients with various symptoms following HPV vaccination showed significant reduction in CBF in the regions associated with the right limbic system [24]. Our present study identified unique brain regions with glucose hypometabolism, some of which matched the regions with decreased CBF, namely the mesial temporal region and the thalamus [24].

Several other PET studies have reported changes in cerebral glucose metabolism in FSS spectrum disorders. Patients with CFS manifest a significant reduction in cerebral glucose metabolism in the medial–frontal cortex and brainstem [16] or a small reduction nonspecifically in the brain [25]. In patients with fibromyalgia, an elevation of glucose metabolism in the limbic system is observed post-treatment [17]. A series of studies on glucose metabolism in these FSS spectrum disorders indicated that the limbic region might be a core region for the development of FSS symptoms.

This one-stop measurement of glucose metabolism may be used to estimate the steady-state level of cerebral function. Combining metabolic steady-state and inflammatory state measurements could allow additional information on the brain milieu. Beside the limbic system, we detected significant glucose hypometabolism in the dorsal part of the brainstem and thalamus. As discussed below, the current cerebral hypometabolism regions in FSS might be linked with increased neuroinflammation because of co-occurrence with the decreased [^18^F]FDG and increased [^11^C]DPA713, suggesting that glucose hypometabolism could be associated with neuroinflammation. At least in the current study, brain volume was not responsible for this hypometabolism because a VBM analysis showed no difference between FSS and age-matched control groups. Thus, cerebral glucose hypometabolism in these regions might be pathophysiologically implicated.

PET imaging of TSPO is widely used to monitor neuroinflammation in neurological and psychiatric disorders [9, 12]. A recent PET study revealed significantly higher [^11^C](R)-PK11195 BP_ND_ in patients with chronic fatigue syndrome than in healthy controls in the thalamus, midbrain, and pons [18]. The pattern of high [^11^C](R)-PK11195 BP_ND_ in these regions is consistent with our findings. Moreover, in our study using [^11^C]DPA713, which is more specific than [^11^C](R)-PK11195 [22], increased [^11^C]DPA713 binding was found in broader regions covering the mesial temporal region, thalamus, and brainstem in FSS.

One possible explanation for the presence of neuroinflammation may be related to the inflammatory cytokines in circulation. One review about patients with CFS described that circulating cytokines, such as tumor necrosis factor-α, interferon-γ, interleukin (IL)-6, and IL-1, are associated with the initiation of inflammation [26]. Patients with fibromyalgia were reported to show higher serum levels of IL-1 receptor antagonist, IL-6, and IL-8 [27]. Hence, inflammatory cytokines may affect the brain regions described above due to cytokine infiltration. Another possible mechanism of neuroinflammation may be stress-induced neuronal deactivation. Indeed, stress sensitizes peripheral as well as central cytokine responses, leading to neuroinflammation and hypothalamic–pituitary–adrenal axis activation [28]. Endogenous cytokine expression and associated cytokine receptor responses are found throughout the brain, including the circumventricular thalamic regions, brainstem nuclei, hypothalamus, and basal ganglia [28]. In any possibility, neuroinflammation in the unique brain regions is an underlying phenomenon responsible for FSS symptoms.

We found unique brain regions with cerebral glucose hypometabolism and neuroinflammation that were associated with key clinical features in patients with FSS. Significant correlations of psycho-behavioral scores with levels of [^18^F]FDG SUVR and [^11^C]DPA713 BP_ND_ were found mainly in the thalamus and hippocampus (Table 3). Although there was a right–left lateralization in regions with significance, the levels of [^18^F]FDG SUVR and [^11^C]DPA713 BP_ND_ in the thalamus and hippocampus on both sides correlated considerably well with psycho-behavioral scores (Table 3). This suggested that thecurrently observed lateralization is not essential in the clinico-bioindex relevance. It is known that the thalamus is important in working memory and processing speed [29], acting as a central monitor for language processing and cortical activity [30, 31]. The circumventricular regions that were highlighted in the present study are associated with neuroendocrine regulation, stress response, immunomodulation, and cognitive regulation [32]. The hippocampus is also associated with language comprehension and processing speed [33, 34]. In the brainstem, the dorsal part, including the locus coeruleus and raphe nucleus, were highlighted in the present study. These nuclei are origins of the noradrenergic and serotonergic systems, subserving autonomic and emotional control, respectively. Taken together, the neuroinflammation-induced cerebral glucose hypometabolism in these peculiar regions may lead to the development of FSS symptoms.

Interestingly, our direct comparison between [^18^F]FDG SUVR and [^11^C]DPA713 BP_ND_ showed a positive correlation in the right hippocampus and amygdala. Since it was reported that TSPO-based PET findings in chronic, but not early, brain disease reflect a proinflammatory state [35, 36], it seems theoretically possible that lower cerebral metabolism couples with higher proinflammatory responses, i.e., [^18^F]FDG SUVR could negatively correlate with [^11^C]DPA713 BP_ND_. Our present result contradicts this hypothesis. Because [^18^F]FDG measurement does not differentiate anaerobic glycolysis, which is predominant in microglia [37], from aerobic glycolysis, the current result suggested that the higher [^18^F]FDG uptake accompanied greater glycolysis in the hippocampus and amygdala, which subserve memory and emotion in patients with FSS. In the present study, a significant reduction in glucose metabolism was found in patients with FSS compared with healthy subjects. Hence, one possible interpretation is that the true amount of glucose metabolism in neurons might be much lower than the observable level that contained glial glucose consumption. One solution may be the use of a new PET tracer for oxidative metabolism in mitochondria. Oxidative metabolism is higher in neurons than in glial cells. Therefore, the new PET tracer could be used to distinguish the neuron-dominant glucose metabolism from neuroinflammation-related glucose consumption [38].

The present study has several limitations. First, the etiology of FSS is unknown. The patients in the current study developed FSS symptoms after different time intervals following HPV vaccination. HPV vaccination might contribute partly to FSS: HPV-treated mice manifest altered behavioral responses and microglial activation in the hippocampus CA1 area [39]. However, our results showing a greater extent of neuroinflammation outside the hippocampus and the development of FSS symptoms irrespective of vaccination [40] suggest that HPV vaccination may not be involved in the development of FSS, rather a need to prepare for development of FSS if one has altered metabolism or neuroinflammation in the thalamo-limbo-brainstem region in the brain. Second, the rs6971 polymorphism poses an issue for second-generation TSPO tracers such as [^11^C]DPA713 [41]. As most Asian people are moderate affinity binders with moderately homogenous pattern of polymorphism [41], our findings in Japanese women may not have been affected by the polymorphism. Third, there was a significant age difference in the [^18^F]FDG study between patients and controls (patients were younger than controls). While there was no significant change in brain volume in the current study from prior data of younger individuals, the previous study indicated that cerebral glucose metabolism is subject to age-related changes, especially in the dorsolateral prefrontal, inferior temporal/fusiform, and primary somatosensory cortices [42, 43]. This suggests that lower glucose metabolism found in the FSS group would have been more remarkable if a strictly age-matched control group was used. Fourth, our sample size was relatively small. Despite this limitation, we were able to show significant differences among groups under a rigorous statistical level.

## Conclusions

Our results show that both cerebral glucose hypometabolism and neuroinflammation were concurrently present in unique brain areas covering the thalamo-limbo-brainstem region in patients with FSS. A close relationship of cognitive impairment with metabolic and inflammatory abnormality in these regions provides better understanding of the clinicopathophysiology of FSS.

## Data Availability

The datasets used and/or analyzed during the current study are available from the corresponding author on reasonable request.

## Abbreviations

AC–PC: Anterior commissure–posterior commissure
BP_ND_: Binding potential
CBF: Cerebral blood flow
CFS: Chronic fatigue syndrome
FSS: Functional somatic syndrome
FWE: Family-wise error
HPV: Human papillomavirus
IL: Interleukin
MNI: Montreal Neurological Institute
MRI: Magnetic resonance imaging
PET: Positron emission tomography
ROI: Region of interest
SD: Standard deviation
SPECT: Single-photon emission computed tomography
SPM: Statistical parametric mapping
SUVR: Standardized uptake value ratio
TAC: Time activity curves
TSPO: Translocator protein
VBM: Voxel-based morphometry
WAIS-III: Wechsler Adult Intelligence Scale-Third Edition
WMS-R: Wechsler Memory Scale-Revised
